# Medication charts and consent to treatment documentation audit in an acute mixed in-patient psychiatry unit in south Manchester

**DOI:** 10.1192/bjo.2021.879

**Published:** 2021-06-18

**Authors:** Madhumanti Mitra, Shahid Hussain, Emma Raynor, Joanna Wong, Jennifer Thom

**Affiliations:** Greater Manchester Mental Health NHS Foundation Trust

## Abstract

**Aims:**

The main aim of this audit was to look at documentation in medication charts in an acute mixed inpatient unit in South Manchester. In addition, we also looked at completion of capacity assessment and consent to treatment forms as appropriate.

**Background:**

Safe prescription, administration and monitoring of medication is key to effective patient care. Due to the busy nature of inpatient hospital wards, errors do unfortunately occur both with the medications, and with the recording of their administration.

We will use a data collection tool to collect data as per standards described in our local GMMH policy. The medication chart will be used as the standard, as this is the current chart that is in use in the Trust.

**Method:**

Data were collected from 31 medication charts for inpatients admitted in the ward between the 5/12/19 to 18/12/19. We captured data from each page of the medication chart that required a record to be made by any staff, including details of prescribing, administration and pharmacist checks. Data were recorded as either Yes/No or NA (Not Applicable). Data were then summarised and analysed using MS excel.

**Result:**

Of the 31 patients, 22 (71%) had a capacity assessment form completed and 16 (52%) had a consent to treatment form completed. From the data analysis, it was clear that there are high rates of completion for the ‘essential’ parts of all prescriptions, including medicine name, dose, route and data. ‘Route’ was only recorded for 40% of prescriptions for depot medicines. Details of the administration of a medicine by a nurse was generally well-completed. For as required medications, all information relating to administration (date, time, dose and given by) were fully completed for 100% of prescriptions. For regular prescriptions however, the administration details were not as well-completed, where date of administration was recorded in 84% of prescriptions and signature in 29% of prescriptions. Unique patient identifiers are well-recorded on Page 1 of the prescription chart, though not maintained throughout the prescription chart. Nature of reaction to an allergy or sensitivity was only recorded in 6 of the 21 patients (29%).

**Conclusion:**

Overall, there were good completion rates for the mandatory parts of the prescriptions. However improvements could be made for prescriptions as well as administration and pharmacy checks. The capacity assessment and consent to treatment forms could be improved upon too. We plan to put the recommendations and re-audit in 3-6 months’ time.

